# A Real Case Study of a Full-Scale Anaerobic Digestion Plant Powered by Olive By-Products

**DOI:** 10.3390/foods10081946

**Published:** 2021-08-21

**Authors:** Antonia Tamborrino, Filippo Catalano, Alessandro Leone, Biagio Bianchi

**Affiliations:** 1 Department of Agricultural and Environmental Science, University of Bari Aldo Moro, Via Amendola 165/A, 70126 Bari, Italy; alessandro.leone@uniba.it (A.L.); biagio.bianchi@uniba.it (B.B.); 2 Department of Biosciences and Territory, University of Molise—Contrada Fonte Lappone, 86090 Pesche (IS), Italy

**Keywords:** biogas production, full-scale plant, olive oil by-products, energetic performance, anaerobic digestion (AD)

## Abstract

The anaerobic digestion plant studied in this paper is one of the first full-scale plants using olive oil by-products. This is a two-stage plant with a power of 100 kWe. Two tests were performed: the first on olive pulp and pitted pomace and the second on biomass consisting of 10% crushed cereal. In both cycles, the retention time was 40 days. The production of biogas was between 51 and 52 m^3^/h, with limited fluctuations. The specific production values of biogas indicate that a volume of biogas greater than 1 m^3^/kg was produced in both tests. The produced biogas had a methane percentage of about 60% and the specific production (over total volatile solids, TVS) of methane was of the order of 0.70 m^3^methane/kg_TVS_. FOS/Alk (ratio between volatile organic acids and alkalinity) was always lower than 1 and tended to decrease in the second digester, indicating a stable methanogenic phase and the proper working of the methanogenic bacteria in the second reactor. The concentration of incoming biomass TPC (total polyphenols content) can vary significantly, due to the seasonality of production or inadequate storage conditions, but all measured values of TPC, between 1840 and 3040 mg gallic acid kg^−1^, are considered toxic both for acidogenic and methanogenic bacteria. By contrast, during the process the polyphenols decreased to the minimum value at the end of the acidogenic phase, biogas production did not stop, and the methane percentage was high.

## 1. Introduction

The production of olive oil is one of the most important agro-industrial activities of the Mediterranean countries and, worldwide, there has been a gradual increase with about 3 million tons produced in 2015. The industrialization of olive oil production has determined the production of huge quantities of vegetation water and/or pomace. Identifying an ecological and economically viable solution for the management and disposal of oil mill waste is one of the main objectives for olive oil-producing countries [1,2,3,4,5,6,7,8].

The vegetation water (OMWW) and the pomace (OMSR) have an acidic pH, high values of chemical demand (COD) and biochemistry (BOD_5_) of oxygen, low nitrogen content, and the presence of lipids and a phenolic fraction [9,10]. The high organic load (45–220 g/L of COD) represents a significant energy potential and would make the olive wastewater one of the most suitable agro-industrial wastes for anaerobic digestion [11,12]. The high content of cellulosic and toxic substances, such as phenols, long-chain fatty acids (LCFA), ethanol, tannins, etc., are considered the main obstacles to the anaerobic digestion of oil wastes; in fact, in addition to being hardly degradable, they also inhibit the activity of the micro-organisms responsible for the process [13].

In [14], by adding different concentrations of the most important phenolic components of the oily waste (tyrosol, oleuropein, caffeic acids, *p*-hydroxybenzoic acids, and protocatechuic acids) to domestic sludge during tests of anaerobic digestion at 35 °C, it was shown that the phenols inhibited or influenced the anaerobic digestion, and therefore the production of biogas, at different concentrations. At concentrations ≥600 mg L^−1^ oleuropein reduced the speed and amount of methane produced; the caffeic, protocatechuic and *p*-hydroxybenzoic acids were inhibitory at concentrations ≥1000 mg L^−1^ and tyrosol at concentrations ≥2000 mg L^−1^; at lower concentrations, the phenols either improved digestion and biogas production or had no effect.

In [15], the possibility of using first pig manure as co-substrate and then previously digested pig manure was evaluated [16]. In the first case, they used an anaerobic up-flow filter fed with pig manure and pomace to form a mixture obtained in gradual increments of 8 to 83% by volume of OMWW. A methane yield was obtained of 0.341–0.349 m^3^/kg COD removed, showing stable operation even at the highest OMWW content of the power supply. In the second case, successive volumetric increases of OMWW ranging from 8% to 91% were mixed with the pig manure digestate. They were then treated through an anaerobic up-flow filter, obtaining greater efficiency and stability of the digester at a concentration of 83% (*v*/*v*) with total removal of COD 73–75% and gas production from 1.7 to 2.1 m^3^/m^3^d, with a yield of 66–68% of CH_4_.

Using two-stage continuously stirred tank laboratory reactors, in [17] a mixture of 55% olive oil wastewater, 40% whey, and 5% (*v*/*v*) of cow manure was used. The effluent was successfully degraded and a methane production rate of approximately 1.35 m^3^ m^−3^ d^−1^ was obtained using an HRT of 19 days. In [18], the anaerobic co-digestion of olive wastes (OMW) with pig manure (PM) was carried out at different mixing ratios in continuous and discontinuous mode. In [19] a useful pre-treatment of OMWW was identified, as a result of laboratory tests, for the removal of COD of 52% and 42%, respectively for aluminium and iron, made with an electrochemical process at pH = 6.0, using aluminium and iron anodes. Similarly, in [20] a combined treatment of electrocoagulation and sedimentation was applied, obtaining the removal of about 70 phenolic compounds, an increase in methane yield of 90%, and a reduction of 33.6% of the soluble COD.

In [21], both the co-digestion with pig manure and the mono-digestion of two-phase process pomace (TPOP) were carried out, at mesophilic conditions, in a two-stage CSTR reactor. The results obtained showed that the co-digestion of TPOP and PM has process stability and a methane production rate greater than mono-digestion. The studies [21,22] subjected the virgin pomace of the two-phase process (OMSW) to a heat treatment, obtaining an increase in soluble COD, a decrease in cellulose and lignin content, and an increase in the maximum methane production rate of 22% compared to non-treated OMSW. Reference [23] obtained a potential increase of 5.6% in methane production compared to non-treated OMSW by ultrasonic pre-treating the two-phase OMSW. However, the net balance between the energy consumed during pre-treatment and the potential energy production gave a negative value. Moreover, chemical pre-treatments based on the addition of NaOH, polyelectrolyte salts (FeSO_4_ FeCl_3_ MnSO_4_), and CaCO_3_ to two-phase pomace led to biogas production values that were twice those obtained with the pomace diluted with water. A reduction of 50% of polyphenols was recorded [24].

Recent studies have experimented at a laboratory scale on the possibility of producing biogas through the anaerobic digestion of 100% of olive oil waste. Methane-forming bacteria can use a limited number of substrates to form methane; acetic acid (AcH) is the best precursor for more than 70% of methane. The other short-chain organic acids, such as propionic and butyric acids, are important substrates in the formation of methane, but they are toxic at certain levels of concentration [25]. Therefore, it is important to know the optimal conditions of the acidogenic phase of the anaerobic process, in order to maximize the production of AcH.

In [26], a laboratory-scale study was carried out on the effect of the organic loading rate (OLR) on the anaerobic acidogenic fermentation of the TPOP in a mesophilic digester (35 °C). The experimental results showed that the optimal value of OLR, for which a maximum production of volatile organic acids (14.5 g L^−1^ expressed as acetic acid) was obtained with a high concentration in acetic acid (57.5% of total concentration), was 12.9 g T-COD/L d noting that the inhibition of the process occurred at OLR above 12.9 of g T-COD/L d. The failure of the process for higher OLR was characterized by a significant decrease in the concentration of acetic acid in the digestate and an increase in the concentration of other volatile acids that can subsequently influence the methanogenic phase.

In a subsequent study, in [27] tests were carried out on the methanogenic phase of an anaerobic digestion process at mesophilic temperatures (35 °C) for the treatment of two-phase pomace (TPOP). The substrate used for the methanogenic phase was the effluent of a hydrolytic-acidogenic reactor operating at the optimal values identified in the previous study, i.e., a volumetric organic load (OLR) of 12.9 g COD L^−1^ d^−1^ at a hydraulic retention time (HRT) of 12.4 days. A high stability of OLR was obtained below 20.0 g of COD L^−1^ d^−1^, justified by the ratio of fatty acids/alkalinity: 0.12 for HRT of 4–6 days. The COD was removed for values between 94.3% and 61.3% and the volatile solids (VS) were removed between 92.8% and 56.1% for the OLR between 0.8 and 20.0 g of COD L^−1^ d^−1^. Likewise, a 43.8% reduction was achieved for the phenolic content. The above results have shown that the two-stage anaerobic digestion process has high stability both in the hydrolytic-acidogenic phase and in the methanogenic one.

In [28], the possible exploitation of the advantages of two-stage anaerobic digestion was examined, on a laboratory scale, for the treatment of an OMWW mixture and of solid olive residues (OMSW), using two semi-continuous digesters in sequence and operating at mesophilic temperature (37 ± 2 °C). The results showed the possibility of degrading the olive oil wastes in co-digestion without dilution and without adding an expensive chemical nitrogen substrate. Methane productivity of 40.17 ± 0.9 L/LOMW was obtained with an OMWW COD level of 196 ± 5 g COD/L co-digested with a 24-day water retention time (HRT) in the first and in the second phase, while the COD removal of 82 ± 0.95% was achieved with an OMW COD level of 196 ± 5 g COD/L co-digested with OMSW at a 24-day HRT in the first phase and 36-day HRT in the second phase, with a phenol removal of 70–78%.

From the foregoing, the scientific results obtained so far have led to the setting of a limit for polyphenols at 600 ppm for the anaerobic digestion, as well as to the design of two-stage industrial plants, fed with biomass consisting of a percentage of olive oil by-products of no more than 30% and no less than 100 kW of produced electric power. Most studies aim to identify process parameters and the complementary substrate that can compensate for alkalinity deficits, phenols inhibition, and nitrogen and micronutrient deficiencies, in order to improve the anaerobic digestion of the oleaginous waste and optimize biogas production.

In this paper, the anaerobic digestion plant studied is one of the first full-scale plants which uses only olive oil by-products. In particular, the results of experimental tests carried out on this plant are shown. The aim was to analyse the real-scale process of anaerobic digestion of by-products of olive oil production, in order to evaluate the differences between the studied case and the above scientific literature data. Moreover, feasibility, process variables, design, and operational parameters are evaluated for this particular anaerobic digestion industrial plant processing olive oil by-products.

The novelty of this study is the industrial-scale evaluation with useful results for industrial plants.

## 2. Materials and Methods

### 2.1. Industrial-Scale Anaerobic Digestion Plant

**Figure 1:**
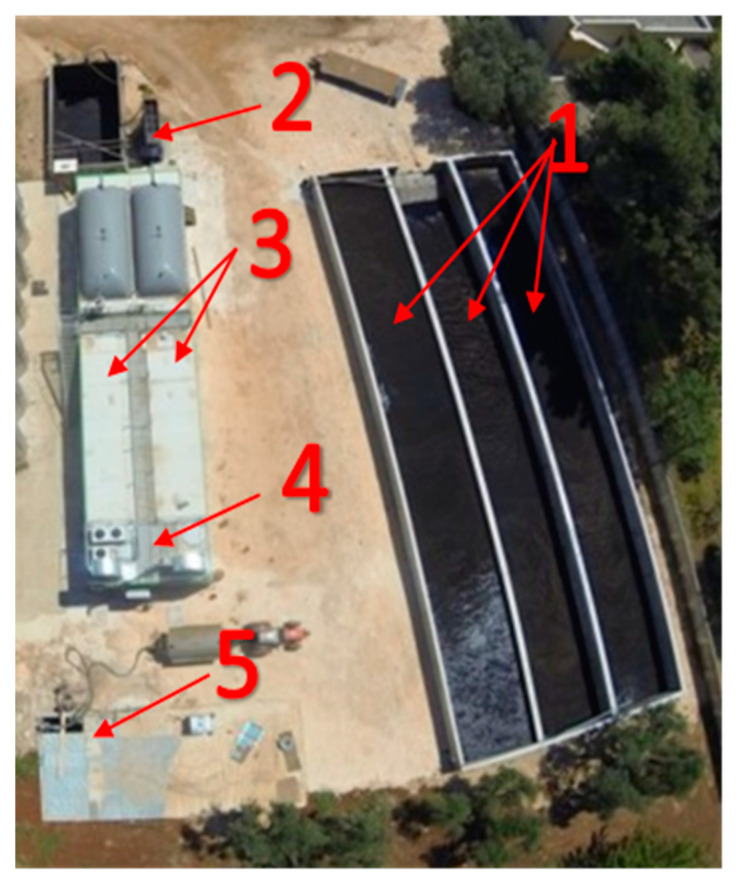
Overview of the full-scale anaerobic digestion plant. (1) biomass storage tanks; (2) loading hopper; (3) plug-in digesters; (4) cogenerator engine; (5) cogenerator biogas line.

The experimental tests were carried out on an industrial-scale anaerobic digestion plant of 100 kWe installed at an olive oil mill company (Frantoio Oleario Domenico Cassese, s.p., Grottaglie, (BR) Italy). The company can produce more than 3000 t per year of pulp that is basically made up of pitted pomace and vegetable water and has about 80% of the moisture used as biomass on the anaerobic plant. The studied anaerobic digestion industrial plant (EUCOMPACT model, Schmack-Biogas Srl, Bolzano, Italy) is a two-stage plant operating in mesophilic conditions, at about 42 °C (Figure 1).

The plant, as reported in Figure 1, consists of:

(a) three open tanks in reinforced concrete for the storage of the pulp and the pitted pomace mixed together with a hopper for automated biomass loading in cycles of present duration;
(b) two plug-in digesters arranged in parallel, each with a capacity of 200 m^3^ and equipped with a rotary reel shaker/mixer; the biomass temperature control is carried out through the circulation of hot water both in jackets on the walls and into the duct made in each rotary reel;
(c) a container, which houses the cogenerator, the heating system of digesters and external users, the gas treatment system, the control and management system, and the substrate pumping system; an underground covered concrete tank for the storage of the digestate.

**Figure 2:**
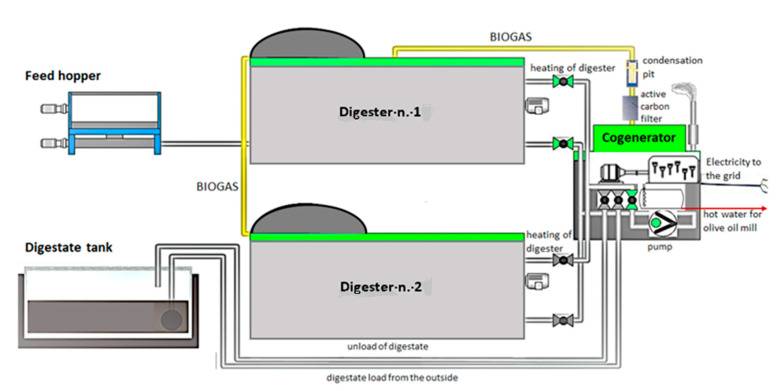
Schematic of the full-scale anaerobic digestion plant. Details of process flow.

A scheme of the plant is reported in Figure 2. The produced biogas is used by the CHP (100 kWe) to generate electricity and heat. The thermal energy of the engine is recovered in the form of hot water, used for the thermostatic control of the digestion system throughout the year, as well as for the needs of the olive oil extraction plant and the households during the winter months. The produced electricity can be used for self-consumption to meet the needs of the digestion system and the surplus is sold to the national electricity grid.

### 2.2. Experimental Set-Up and Operation

The retention time of the treatments is 40 days. In the first digester the hydrolytic phase takes place with an acid-forming time of about 20 days, and then in the second digester the methanogen phase takes place with an acid-forming time of a further 20 days to complete the 40-day treatment. Two different compositions of biomass with minimal differences have been considered for the experimental tests:

(a) (Test A) basically, pulp and pitted olive pomace;
(b) (Test B) pulp pitted olive pomace and wheat bran shorts in the amount of 10% of the total powered biomass. The wheat bran was added to the first reactor immediately before the inlet.

In both tests, an amount of urea equal to 0.75% of the feed flow and a powder formulation of micronutrients: cobalt, iron, selenium, manganese, molybdenum, and zinc in a quantity of 2 kg of microelements every 100 days, were added. The biomass (test A and test B) is representative of what is usually used during the year in a typical plant. In some periods of the year, additions are made with waste from the milling plants to ensure a constant flow of biomass to the plant. For this reason, the composition of test B was also chosen and experimentally studied. The evolution of digestate and biogas characteristics were investigated in samples taken according to the experimental design, thus during loading (A1 and B1), after 10 days (A2 and B2), after 20 days (A3 and B3), and after 40 days of treatment (A4 and B4). Three samples of 5 kg of biomass were taken for each sampling point. The analyses were repeated five times for each sample.

### 2.3. Analytical Methods

The following chemical analyses were performed on the samples of biomass and digestate taken, in order to monitor the fermentation process in progress within the plant.

#### 2.3.1. pH

pH of the biomass samples was tested using the Crison pH meter device—GLP pHmeter 32, Electrodia, with three membranes for turbid liquids, and calibration change pH 4.00–7.01–10.0.

The instrument was calibrated with the calibration solutions and then the measurement was carried out.

#### 2.3.2. Total Solids and Total Volatile Solids

The total solids and total volatile solids contents of the biomass were determined according to the IRSA CNR (1984) [29] and IRSA CNR (2003) [30] methods. The ISCO NSV 9035 air circulation thermostat and the NOBERTHERM muffle temperature up to 1100 °C were used. The sample was placed in a thermostat to determine the loss of water at 105 °C and the dry residue (total solid) was then placed in a muffle at 600 °C for the determination of the ashes. The difference between the total solids at 105 °C and the ashes at 600 °C, constitutes the volatile solids (SSV).

#### 2.3.3. Total Polyphenols

The total polyphenol contents were determined by a colorimetric method with Folin–Ciocalteu reagent using the Perkin Ellmer lambda 2 UV/VIS double beam spectrophotometer. The method is based on the “polyphenol-reactive colorimetric reaction of FOLIN CIOCALTEUS”. The reagent was from Sigma–Aldrich. The reagent contains phosphotungsten and phosphomolybdenum: in a basic environment the phosphorus and molybdenum form a blue complex with the polyphenols; the intensity of the color (in the linear range described by Lambert Beer’s law) is proportional to the concentration of polyphenols. The calibration curve was constructed with known quantities of polyphenol: gallic acid was used as the standard polyphenol. Then, following the same procedure, the polyphenol/reagent colour was developed on the sample to be analysed and read on the spectrophotometer at 760 nm. Particular attention was paid to the solubilization of the polyphenols present in the sample: this operation lasted one night and is described in the working method. The device that has memorized the calibration curve has given the value in mg/kg of polyphenols expressed as gallic acid.

#### 2.3.4. Total Nitrogen

Total nitrogen concentration was analysed using the VELP DK6 mineralizer equipment and the VELP UDK 140 distiller. The organic nitrogen compounds are transformed into ammonia: this transformation takes place in an acid environment with a catalyst and in hot conditions. The following reagents were used:

- concentrated sulfuric acid;
- copper sulphate + potassium sulphate (ratio 1:3): catalyst;
- boric acid;
- 0.1 N hydrochloric acid;
- 30% sodium hydroxide;
- Tashiro indicator (bromcresol green + methyl red).

In the mineralizer at 420 °C all the organic nitrogen was transformed into ammoniacal nitrogen. After cooling, the solution was alkalized and all the ammonia nitrogen was distilled in a stream of steam. The ammonia was absorbed by a solution of boric acid (40 gr/L) and the absorbed ammonia was titrated with 0.1 N hydrochloric acid. The hydrochloric acid equivalents used were equivalent to the ammonium equivalents in the solution. The titrated ammonium was the sum of the ammonium present in the solution before digestion and the ammonium that was formed following digestion by transformation of organic nitrogen into ammonium.

#### 2.3.5. Ammoniacal Nitrogen

Ammoniacal nitrogen concentration was analysed using the VELP UDK 140 distiller equipment according with the AOAC 973.48, 1973 [31]. The following reagents were used:

- 0.1 N hydrochloric acid;
- 30% sodium hydroxide:
- boric acid;
- 30% sodium hydroxide;
- Tashiro indicator (bromcresol green + methyl red).

The VEL UDK 140 apparatus as for total nitrogen was used. The organic nitrogen was calculated as difference between total nitrogen and ammonia nitrogen.

#### 2.3.6. Metals and Microelements (Cu, Fe, Na, Mn, Zn, K)

The EPA 6010b [32] and EPA 3052 [33] were used for the metals and microelements. The Perkin Elmer Optima 8000 ICP-OES spectrometer was used with a Milestone Strt D microwave mineralizer. Standard solutions of the individual elements were used.

The samples were mineralized in an acid medium in the microwave digester; then after suitable dilution with double distilled water it was injected into ICP to directly measure the concentration in mg/L. ICP memorized the calibration curves of each element and then processed the final result.

#### 2.3.7. Ratio between Volatile Acid and Alkalinity (FOS/TAC)

The method used to estimate the biochemical biogas potential was based on a volumetric test, which considered the displacement of a liquid into gas to measure the biogas production [34]. A sample of fermentation substrate was titrated by 0.1 N of sulfuric acid solution (H_2_ SO_4_) up to pH 5.0 to calculate the TAC value, expressed in mg/L of calcium carbonate (CaCO3). A centrifuge rotating at 4000 rpm was used as apparatus. An aliquot of material was centrifuged at 4000 rpm × 5 min and 20 cc aliquot of the supernatant was titrated with 0.1N sulfuric acid. Titration took place in two steps:

- first step titration between initial pH at pH 5.0 A = mL 0.1 N sulfuric acid;
- second step titration from 5.0 to 4.4B = mL 0.1 N sulfuric acid.

An empirical formula was developed that expressed the ratio of acids and alkalinity:

FOS = (B × 1.66 − 0.15) × 500

TAC = A × 250

FOS/TAC = factor that indicates the running stability of the reactor

A well-functioning anaerobic digester has a FOS/TAC value of 0.25–0.35. All analyses were performed in triplicate. The analytical value was presented as the arithmetic mean of the three values.

### 2.4. Plant Performance Evaluation

To evaluate the plant performances, the following technical parameters were acquired by the software BIOWATCH of the company Schmack-Biogas s.r.l. based on PLC Siemens with the relative software WinCC:

(a) feed rate, measured using an electromagnetic flowmeter, Endress+Hauser Proline Promag 55S (DN 150);
(b) the average temperature inside the digesters, measured at the inlet (point 1) and the outlet (point 2) of each digester by means of chromo-constantan thermocouples;
(c) percentage of methane, measured using an AWITE continuous analyser (AWIECO model), to which the biogas is sent before and after the desulfurization treatment at 4-h intervals;
(d) engine cooling water temperature, measured by chromium-constant thermocouples positioned at the exhaust of the cooling system:
(e) the energy produced (EP) and energy fed into the grid (ER), detected by the acquisition system installed by the GSE (energy services manager);
(f) energy for self-consumption (EA), obtained from the difference EA = EP − ER.

The previously mentioned management software helps the operator in modifying the daily flow rate of the biomass in the plant, basing it on the measurement of the gas volume in the digester’s gasometers and changing the operating time of the screw pump (nominal flow rate 5.0 m^3^/h). The biogas flow rate was also measured using a Siemens flowmeter mod. SITRANS FC430 is equipped with a SITRANS FCT030 transmitter. The results are represented as a trend over time of the measured parameters of the individual tests conducted.

### 2.5. Statistical Analysis

The statistical analysis of the data was based on the calculation of the mean and the standard deviation, based on the nature of the individual variables; the experimental data were analyzed using the ANOVA test, Tukey’s HSD for multiple range test (*p* < 0.05), and Student’s *t*-test, using the MATLAB^®^ statistics toolbox (The Mathworks Inc., Natick, MA, USA).

## 3. Results and Discussion

### 3.1. Impact of the Operating Conditions on Energy Performance and on Digestate Composition

Table 1, Table 2, Table 3, Table 4 and Table 5 show results respectively related to:

(a) the operating conditions and the main plant performance parameters in both test periods;
(b) the chemical analyses on the evolving biomass;
(c) the main energy performance parameters of the plant and the characteristics of digestate with particular reference to agronomic parameters.

The total solids (TS) of the incoming biomass, equal to 15.18% in the first period and 16.55% in the second period (Table 2 and Table 3), show that, in this case, the digestion of the organic load can be classified as wet digestion. The temperature is substantially constant at 42.5 °C for all the hydraulic retention time (HRT) (Table 1).

The specific organic load is between 5.33 and 5.54 kg TVSm-3d^−1^. It leads to specific biogas production between 1.10 and 1.17 m^3^ biogas/kg TVS (total volatile solids) and a specific methane production between 0.66 and 0.70 m^3^ methane/kg TVS (Table 1). These results show that, in both digesters, there are optimal microclimate conditions for mesophilic anaerobic digestion. The mixing/heat exchange system and the temperature control are efficient, allowing continuous biogas production with quite a high methane percentage, of more than 59% (Table 1), despite the fact that the specific organic load values are close to the critical ones, especially for wet digestion [35]. The mean biomass feed rate is equal to 7.99 m^3^/24h in test A and 7.22 m^3^/24h in test B (Table 1). Therefore, there is a tendency to feed the plant during the two tests with comparable flow rates, but slightly higher rates when using the biomass with a lower concentration of total solids and volatiles (Table 2 and Table 3).

In fact, the biomass of test A (Table 2) presents mean values of 15.18% for total solids and 13.87% for volatile solids compared to, respectively, 16.55% and 14.79% of test B (Table 3). It follows that, regardless of the addition of a reduced quantity of wheat bran shorts, the solids content of the mixture of pulp and pitted olive pomace tends to increase, both depending on the different characteristics of by-products coming from the olive oil mill and on the stratification of the biomass in the storage tank. Standardization of the solids content could be achieved with an adequate mixing of the biomass during storage, taking into account, however, that no anoxic conditions occur at this stage because volatile solids do not reduce (Table 2 and Table 3).

**Figure 3:**
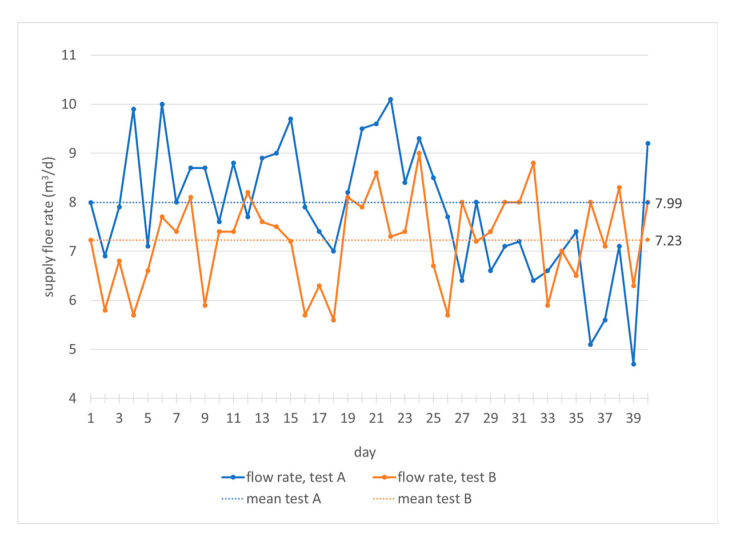
Monitoring of feeding biomass flow rate in the digesters during the hydraulic retention time.

**Figure 4:**
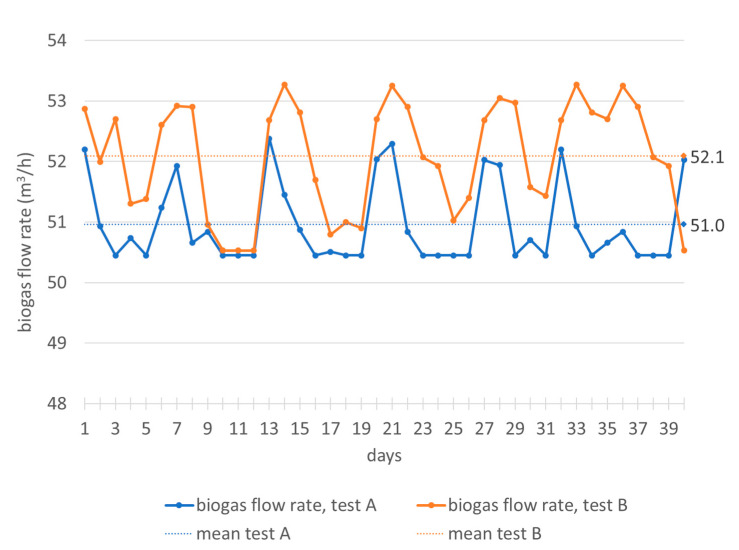
Monitoring of biogas flow rate in the digesters during the hydraulic retention time.

It can be noticed (Figure 3) that in test A, the feeding flow rate has been varied between 4.9 and 10.2 m^3^/d and in test B between 5.7 and 9.1 m^3^/d, with frequent variations during the 24 h. In any case, changes in feed flow correspond to variations in biogas production that do not exceed 3 m^3^/h in both tests (Figure 4). The correlation between the flow variations and the biogas production is not immediate, because the plant processes organic load values not always close to the maximum rate. Sometimes the minimum feeding flow values correspond to the maximum values of biogas production and the response of the plant is quite immediate; in other cases, there is an opposite trend. Anyway, as mentioned, the biogas flow rate varies little during both tests (Figure 4).

Therefore, the flow rate represents an operational parameter useful for managing, in a short time, the specific organic load in the digester and the production of biogas, even for this particular biomass whose antioxidant content could inhibit or slow down the plant’s output. After a deeper analysis of the mean values (Table 1) and the punctual values (Figure 3), it can be highlighted that with lower values of feeding flow rate, higher flow rates of biogas are obtained. In particular, with higher volatile solids content (Table 2, test B), the organic volumetric load decreases, the biomass flow rate reduces and biogas production increases to almost 1 m^3^/h (Table 1, test B).

On the other hand, in this specific case, the changes in flow rate could be useful to maintain stable production of biogas and methane, due to variations in the organic load of the inlet biomass. This, of course, could be a limit of the treated biomass, whose volatile solids content cannot be balanced with a suitable mixing with other organic mixtures. In particular, the studied biomass is not a homogeneous mixture due to the seasonality of the production and the presence of a solid phase (pulp and pitted pomace) and a liquid phase (vegetation water). These two components have a different composition that is variable over time [1,2,3,4], so they should be stored in separate tanks and properly mixed both during storage and before being sent to the digester.

### 3.2. Plant Performance

**Figure 5:**
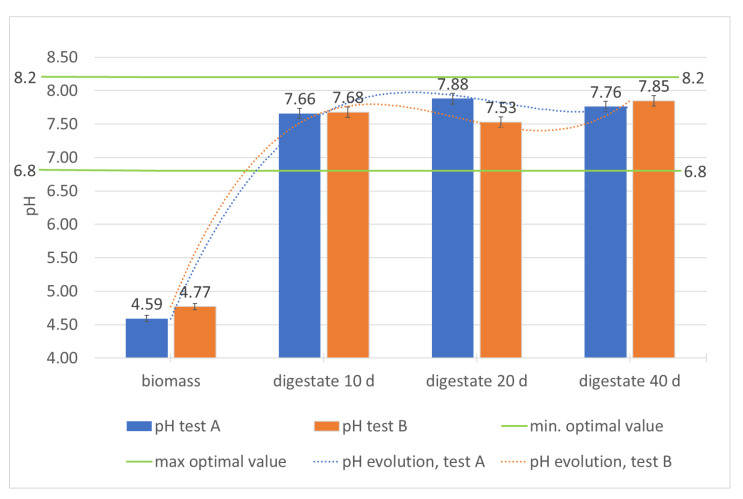
Monitoring of pH in the digesters during the hydraulic retention time.

**Figure 6:**
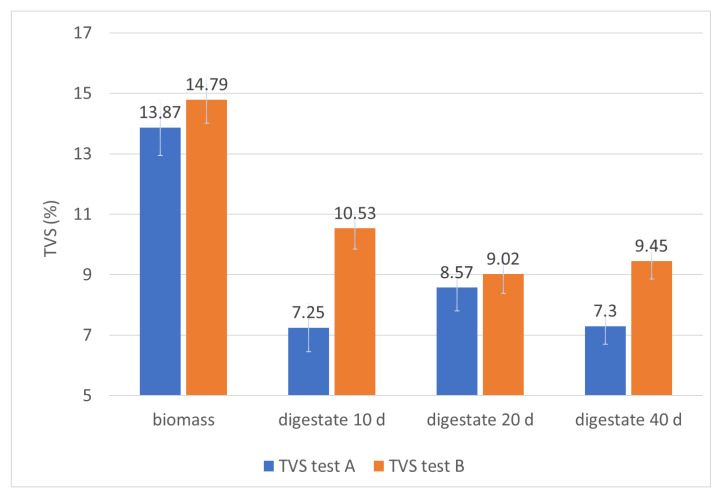
Monitoring of TVS in the digesters during the hydraulic retention time.

**Figure 7:**
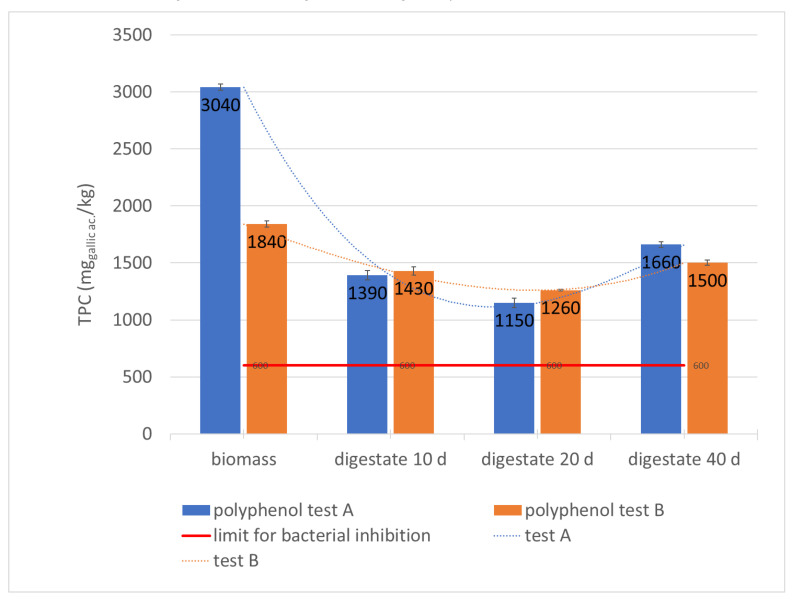
Monitoring of TPC in the digesters during the hydraulic retention time.

The performance of the plant, in relation to the chemical-physical characteristics of the biomass, can be further evaluated by the trend of pH, TVS, and total polyphenols content (TPC) during the residence time of the biomass in the plant, (Table 2; Figure 5, Figure 6 and Figure 7).

The trend of the pH (Table 2; Figure 5) and of the ratio between volatile organic acids and alkalinity (FOS/Alk) shows the remarkable stability of the process. The FOS/Alk ratio (Table 2) is particularly indicative: it is always lower than 1 and tends to decrease significantly in the second digester, showing that the methanogenic phase is quite stable and the probable evolution of the bacterial population towards the methanogenic component occurs in the second reactor. At the same time, starting with pH values of 4.59–4.77 (Table 2; Figure 5) that characterize the incoming biomass, optimal pH values are reached for the production of methane since the acidogenic phase, which occurs in the first digester. Therefore, the biomass pH, the acid at the entrance of the first reactor, tends to neutrality just after 10 days of processing and remains almost constant (except for very small variations) throughout the remaining processing period. The final values of both tests, 7.76 and 7.85 respectively (Table 2; Figure 5), are largely compatible with the requirements related to digestate distribution on soil [35].

The fixed residue (STF) remains significantly unchanged between the incoming biomass and the final digestate (Table 2: samples A4 and B3). In fact, it changes from 1.31% to 1.76% at the beginning of both test A and test B (Table 2), then at the exit of the first reactor is 1.43% for test A and 1.47% for test B (Table 2) and at the output of the second reactor is 1.5% and 1.33% for the tests A and B respectively. This explains why the process, taking place correctly, tends to consume the organic substance, so the relative quantities of the ash do not increase. In fact, the total volatile solids (TVS), passing from the biomass entering the final digestate, decrease from 13.87% to 7.3% in the first test and from 14.79% to 9.45% in the second test (Table 2; Figure 6), suffering a reduction of 47.37% in the first test and 36.11% in the second test. This confirms that in both cases an effective biogas conversion of the organic substance was obtained, as well as a significant reduction of the organic pollutant load of the biomass. However, it should be specified that a more effective metabolic activity of the anaerobic bacteria on a mixture consisting exclusively of pitted pomace and olive pulp is related to a greater presence of aggregates in the biomass of the second test, which leads to lower values of the metabolized organic load.

Both in the first and second tests, inhibitory substances, such as polyphenols and ammonia, are present at concentrations greater than or equal to the levels of toxicity reported in the literature for these molecules [36]. Ammonia tends to increase significantly during the digestion of biomass in both tests, both in the first and second reactor, reaching values >2000 mg kg^−1^: 2079 mg/kg in the first test and 3515 mg/kg in the second test. This increase may also be due to the addition of urea which, in this specific case, could be further reduced. In fact, in these tests the inlet carbon/nitrogen ratio (Table 2) in the biomass is balanced but the excess of carbon compared to nitrogen is easily verifiable in the olive oil wastewater, especially as regards the pomace [1]. The rebalancing of the C/N ratio would produce better effects if waste materials with high protein content are used instead of urea, i.e., vegetable waste from legumes, slaughter by-products, pooled feces, etc.

This result can also be attributed to the efficiency of the adopted mechanical solutions, which allow both efficient heat exchange and high homogenization of the biomass. Therefore, the microclimatic conditions suitable for the development of the methanogenic bacterial population are maintained throughout the fermenting biomass, and the pH is also kept below the toxicity values (Table 2). If the process had developed into a thermophile, the found values of ammonia would have inhibited bacterial activity [36].

The reduction of inhibition phenomena is also attributable to the particular fluid dynamics of the system, similar to a double-stage plug flow where separation of two zones is established:

(a) the first section, in which the acidogenic reactions prevail and there is a partial demolition of the polyphenols because the acidogenic microorganisms are not very sensitive to the inhibitors;
(b) the final section, where methanogenic reactions prevail, by means of methanogenic bacteria that are very sensitive to polyphenols that, in this process, are degraded enough during the acidogenic section (Table 2; Figure 7).

This is clearly highlighted by polyphenols variation (Table 2; Figure 7), from samples A1 and B1 corresponding to the incoming biomass (respectively 3040 mg ac. gallic kg^−1^ and 1840 mg ac. gallic kg^−1^) and samples A3 and B2: during the digestion process, the TPC concentration decreases significantly to their minimum value of 1150 mg ac. gallic kg^−1^ and 1260 mg ac. gallic kg^−1^ (respectively A3 and B2 corresponding to the biomass at the end of the acidogenic phase). Subsequently, during the methanogenic phase, it undergoes a significant increase reaching values of 1660 mg ac. gallic kg^−1^ and 1500 mg ac. gallic kg^−1^ respectively in the A4 and B3 samples corresponding to the final digestate. These data show that the concentration of incoming biomass polyphenols can vary in full-scale plants processing only olive oil waste, due to the seasonality of production or inadequate pomace storage conditions. The increase in concentration between samples A3–A4 and B2–B3, or between the digestate leaving the first digester and the final digestate, can be due to further degradation of the organic substance during the methanogenic phase, helping the solubilization of polyphenols that were still complexed in other structures. Furthermore, according to the literature, the concentration reached in the second stage should be the methanogenic one [14]. However, on the other hand, it is clear that the process takes place regularly with a relevant biogas yield and methane percentage that indicates that bacteria with greater resistance to polyphenols developed within the digesters.

More precisely, in the second reactor methanogenic bacterial strains were selected that became resistant to polyphenols. Finally, they also help solubilization, confirming the slight TPC increase, a phenomenon that can occur in fermentation at full scale [37]. It is not to be excluded that methanogenic bacteria that degrade polyphenols have been selected, removing them from the substrate, but this possibility is less hypothetical because, in this case, the polyphenols would decrease.

The most relevant energetic result is the appreciable specific production of biogas (SGP): 1.10 m^3^ biogas/kgTSV and 1.17 m^3^ biogas/kg TSV, respectively for the first and second test (Table 3). The percentage of methane is just under 60% with a specific production (SMP) of 0.66 m^3^ methane/kg TSV for the first test and 0.70 m^3^ methane/kg TSV for the second test (Table 3). The cogeneration plant produces thermal energy as water at 87–88 °C and electric power ranging between 95.66 kWh and 97.78 kWh. The contract with the electricity supplier stipulates that the company has to use the produced electricity in the anaerobic digestion and cogeneration plant with a minimum rate of 10%; the remaining electricity can be sold to the electricity supplier at an appropriately fixed tariff. If the electricity for self-consumption is less than 10%, the non-consumed energy cannot be sold at the fixed tariff but at a lower one. In the tests carried out, the self-consumption energy is 10.16–11.45 kWh (Table 3) equal to 10.6–11.7% of the electricity produced; however, the condition of not being able to sell all the energy produced by applying the same tariff could be a limit for the company in the economic balance of the energy recovery.

The biomass flow rate, compared to that of digestate (Table 1), is reduced by 28% for the first test and by 25% for the second test, mainly due to the decrease in volatile solids. Therefore, the biomass is concentrated during the process and, even if the digestate is still rather liquid, it has the advantage of being sufficiently stable to be disposed as such on agricultural soil, respecting the limits set by Italian law on agricultural use of olive oil wastewater: 60–80 m^3^/ha. Table 4, in particular metals and microelements content in the final digestate samples, shows that they do not exceed the limits set by the Italian legislation for digestate agronomic use.

The C/N ratio of inlet biomass is optimal (Table 2: 30.9–29.7 respectively for the first and the second test) and C/N final values (Table 4: 8.8–9.5 respectively) show that the discharged digestate can be considered an excellent base of the starting mixture in a composting process, after appropriate dehydration up to 50%. Therefore, it is possible to recover the digestate as part of a balanced mixture to obtain, in a suitably designed composting plant, a commercially viable fertilizer with high agronomic potential.

## 4. Conclusions

This study has been carried out on one of the few full-scale anaerobic digestion plants in the world that processes only olive pomace. The results of the experimentation show the possibility of feeding an industrial anaerobic digestion plant exclusively with olive oil effluents and the performance of the studied plant can be considered comparable with the data of other full-scale plants fed with biomass made up of other types of more tested organic matrices. The results show very appreciable specific biogas production with a high percentage of methane.

The results also show that, for a plant that has to process only olive oil by-products, it is necessary to adopt technology solutions that allow reducing the feed flow rate of the incoming biomass and its stratification, considering that, in this type of by-product, the total input solids are variable during the processing time.

The chemical-physical analyses show optimal values of the process stability indexes and of the parameters that define the regularity of the kinetics of anaerobic digestion. In this particular by-product, inhibitory compounds typical of the composition of the oleaginous by-products, such as polyphenols, in the incoming biomass are found in concentrations higher than the levels of toxicity reported in the literature for these molecules. Nevertheless, the performance of the plant validates the hypothesis that, in the second reactor, methanogenic bacterial strains were developed. These microorganisms adapt to become resistant to polyphenols and even facilitate their solubilization, even if it is not excluded that they are also methanogenic bacteria that degrade polyphenols, thus removing them from the substrate.

The treated biomass undergoes a concentration in the plant and the digestate is still rather liquid but with the advantage of being able to be disposed of in quantities lower than those that characterize the by-products of the company in such form. In this form, it has remarkable agronomic properties, in terms of nutritive elements and C/N ratio. The highlighted agronomic potential could be further enhanced with a suitable composting process that would allow a quality imprint to be obtained with a high commercial value.

From the above, it can be concluded that in a correct future evaluation of the economic feasibility of an investment for the recovery of oil by-products with an anaerobic digestion plant, the use of self-produced electric energy and the sale of the excess rate to the network manager should not be considered the only factors of return. If, in fact, we limit ourselves to these two items, the investment could be impracticable for electric power close to or less than 100 kW, especially if the energy users are seasonal, as in the oil industries, and the sales tariffs managers are not particularly convenient, according to the approach taken by the latest national legislation. It should also be considered that the energy recovery of olive oil by-products also allows a significant reduction of their disposal costs, the possibility of selling the pits, as well as the digestate as a quality improver. Moreover, the mill can take advantage of the benefit of the environmental commitment in primary production that, with the same quality, would allow the selling prices of produced olive oil to rise even more.

## Figures and Tables

**Table 1:** Main operating conditions and performances of the full-scale anaerobic digestion plant-to-energy plant.

Main Operating Conditions	Test (A)	Test (B)
HRT (days)	40	40
Feeding flow rate (m^3^/d)	7.99 ± 1.60	7.22 ± 0.98
OLR (kg_TVS_/m^3^ d^−1^)	5.54	5.33
Temperature (°C)	42.39 ± 0.26	42.23 ± 0.35
Biogas production (m^3^/h)	50.96 ± 0.67	52.09 ± 0.92
SGP (m^3^_biogas_/kg_tvs_)	1.10	1.17
Methane (%)	59.95 ± 1.55	59.89 ± 3.17
Methane flow rate (m^3^/h)	30.55	31.20
SMP (m^3^_methane_/kg_TVS_)	0.66	0.70
Final digestate flow rate (m^3^/h)	5.75	5.41

HRT: hydraulic retention time; OLR: volumetric organic loading rate; SGP: specific biogas production; SMP: specific methane production.

**Table 2:** Results of the chemical analyses of the evolving biomass of Test A.

Chemical Parameters	Test (A)			
	A1	A2	A3	A4
pH	4.59	7.66	7.88	7.76
TS (%)	15.18 ± 1.26 a	8.73 ± 0.67 b	10 ± 0.89 b	8.80 ± 0.58 b
STF (%)	1.31 ± 0.12 a	1.48 ± 0.09 a	1.43 ± 0.15 a	1.5 ± 0.20 a
TVS (%)	13.87 ± 0.92 a	7.25 ± 0.80 b	8.57 ± 0.77 b	7.3 ± 0.60 b
TN (mg kg^−1^)	2598 ± 78 c	5052 ± 96 a	4632 ± 84 b	4781 ± 79 b
ON (mg kg^−1^)	2360 ± 45 d	3488 ± 80 a	2926 ± 78 c	3069 ± 92 b
N-NH_3_ (mg kg^−1^)	289 ± 35 c	1900 ± 87 b	2032 ± 56 a	2079 ± 63 a
C/N	30.9	8.3	10.7	8.8
TPC (mgagallic ac. kg^−1^)	3040 ± 28 a	1390 ± 41 c	1150 ± 41 d	1660 ± 23 b
FOS/Alk	-	0.327 ± 0.003 a	-	0.185 ± 0.001 b

TS: total solids; STF: fixed residue; TVS: total volatile solids; TN: total nitrogen; ON: organic nitrogen; N-NH3: ammoniacal nitrogen; C/N: carbon-to-nitrogen ratio; TPC: total polyphenols content; FOS/Alk: Ratio between the volatile organic acids and alkalinity. A1: inlet biomass; A2: digestate after 10 days; A3: digestate after 20 days; A4: digestate after 40 days. Different letters in rows for each test denote statistically significant difference among means (*p* < 0.05).

**Table 3:** Results of the chemical analyses of the evolving biomass of Test B.

Chemical Parameters	Test (B)			
	B1	B2	B3	B4
pH	4.77	7.68	7.53	7.85
TS (%)	16.55 ± 1.10 a	12.4 ± 1.15 b	10.49 ± 0.41 c	10.78 ± 0.77 bc
STF (%)	1.76 ± 0.28 a	1.55 ± 0.30 a	1.47 ± 0.31 a	1.33 ± 0.32 a
TVS (%)	14.79 ± 0.77 a	10.53 ± 0.31 b	9.02 ± 0.64 c	9.45 ± 0.59 bc
TN (mg kg^−1^)	2875 ± 45 d	7432 ± 54 a	6497 ± 98 b	5749 ± 113 c
ON (mg kg^−1^)	2640 ± 87	4970 ± 53	4416 ± 76	2854 ± 87
N-NH_3_ (mg kg^−1^)	286 ± 53 d	2154 ± 77 a	2527 ± 88 b	3515 ± 63 c
C/N	29.7	7.8	8	9.5
TPC (mgagallic ac. kg^−1^)	1840 ± 27 a	1150 ± 23 d	1260 ± 7 c	1500 ± 23 b
FOS/Alk	-	0.375 ± 0.001 a	-	0.215 ± 0.001 b

TS: total solids; STF: fixed residue; TVS: total volatile solids; TN: total nitrogen; ON: organic nitrogen; N-NH3: ammoniacal nitrogen; C/N: carbon-to-nitrogen ratio; TPC: total polyphenols content; FOS/Alk: Ratio between the volatile organic acids and alkalinity. B1: inlet biomass; B2: digestate after 10 days; B3: digestate after 20 days, B4: digestate after 40 days. Different letters in rows for each test denote statistically significant difference among means (*p* < 0.05).

**Table 4:** Energy performances of the full-scale anaerobic digestion plant and cogeneration plant.

Energy Performances	Test (A)	Test (B)
SGP (m^3^_biogas_/kg_TVS_)	1.10	1.17
Methane (%)	59.95 ± 1.55	59.89 ± 3.17
SMP (m^3^_methane_/kg_TVS_)	0.66	0.70
Produced Energy (kWh)	95.66	97.78
Energy fed into the electricity grid (kWh)	85.50	86.33
Energy for self-consumption (kWh)	10.16	11.45
Water cogeneration T max (°C)	88.07 ± 2.30	87.46 ± 3.10

SGP: specific production of biogas; SMP: specific production of methane.

**Table 5:** Characteristics of digestate.

Parameters	Test (A) Sample A4	Test (B) Sample B4	Limit Value (D.L. 75/2012, D.M. 26 May 2015
pH	7.76	7.85	6–8.5
TVS (%)	7.30 ± 0.60	9.45 ± 0.59	/
C/N	8.8	9.5	<25
Organic carbon (%)	4.2	5.5	30
Total nitrogen (mg kg^−1^)	4781 ± 79	5749 ± 113	/
Downloaded volume (m^3^/d)	5.75	5.41	/
Reduction in volume (%)	28	25	/
Cu (mg kg^−1^)	75 ± 2	67 ± 2	230
Fe (mg kg^−1^)	140 ± 3	141 ± 3	/
Na (mg kg^−1^)	395 ± 11	384 ± 10	
Mn (mg kg^−1^)	12.0 ± 0.5	9.7 ± 1.1	/
Zn (mg kg^−1^)	25 ± 2	19 ± 1	500
K (mg kg^−1^)	5100 ± 12	5850 ± 13	/

TVS: total volatile solids; C/N: carbon-to-nitrogen ratio.

## Data Availability

Not applicable.

## Abbreviations

AcH	Acetic acid
BOD5	Biochemical Oxygen Demand in 5 days
CHP	Combined heat and power
COD	Chemical Oxygen Demand
CSTR	Continuous Stirred-Tank Reactor
HRT	Hydraulic Retention Time
LCFA	Long Chain Fatty Acids
OLR	Organic Loading Rate
OMSR	Olive Mill Solid Residue
OMW	Olive Mill Waste
OMWW	Olive Mill Waste Water
ON	Organic nitrogen
PM	Pig Manure
SGP	Specific production of biogas
SMP	Specific production of methane
STF	Fixed residue
TN	Total nitrogen
TPC	Total Polyphenols Content
TPOP	Two-Phase Process Pomace
TS	Total solids
TVS	Total volatile solids
FOS	Volatile organic acids
FOS/Alk	Ratio between volatile acids and alkalinity
FOS/TAC	Ratio between volatile organic acids and total inorganic carbon
VS	Volatile Solids
